# Adherence to alcohol consumption-related recommendations and predictors of heavy episodic drinking among patients with NCDs during the COVID-19 pandemic

**DOI:** 10.1371/journal.pone.0321577

**Published:** 2025-04-23

**Authors:** Muluken Basa, Jan De Vries, David McDonagh, Catherine Comiskey

**Affiliations:** School of Nursing and Midwifery, Trinity College Dublin, The University of Dublin, Dublin, Ireland; University of Hull, UNITED KINGDOM OF GREAT BRITAIN AND NORTHERN IRELAND

## Abstract

**Introduction:**

Managing non-communicable diseases (NCDs) requires adherence to lifestyle recommendations like a healthy diet, regular exercise, smoking cessation, and limiting alcohol intake. The COVID-19 pandemic introduced barriers to maintaining these habits, including limited healthcare access, increased stress, and reduced physical activity. This study assessed adherence to lifestyle recommendations, with a focus on heavy episodic drinking (HED), among NCD patients during the pandemic in Arba Minch, Ethiopia, to identify areas for public health intervention.

**Method:**

A cross-sectional study was conducted among 310 randomly selected NCD follow-up patients at Arba Minch General Hospital. The data was collected using the WHO STEPS and Coronavirus Anxiety Scale (CAS) tool from March 1 to April 30, 2022. Data analysis included both descriptive and inferential statistics (bivariate analyses and multivariable logistic regression). Confounding variables were identified and controlled for to ensure result accuracy.

**Results:**

Adherence to lifestyle recommendations was found to be low, at just 16.1% [n=50, 95% Confidence Interval (CI) (12.5–20.6%)]. The prevalence of HED was 12.6%, with a higher prevalence among males (18.4%) compared to females (7.4%). Recent alcohol consumption was reported by 29.0% of participants, and among these, 43.3% engaged in HED. Factors significantly associated with HED included male gender (Adjusted Odds Ratio (AOR) 2.63, 95% CI 1.11, 6.24), higher education level (AOR 2.91, 95% CI 1.11, 7.58), and current tobacco use (AOR 6.36, 95% CI 1.62, 25.04). Healthcare disruptions due to COVID-19 (AOR 3.28, 95% CI 1.16, 9.26) and COVID-19-related anxiety (AOR 1.29, 95% CI 1.06, 1.56) were also linked to HED.

**Conclusion:**

The study revealed low adherence to lifestyle recommendationsand significant prevalence of HED among NCD patients during the pandemic. Associations between HED, healthcare disruptions, and anxiety highlight the critical role of mental health and healthcare access in risky behaviors. Targeted public health interventions are essential, including community-based alcohol reduction programs, improved mental health support, and stronger healthcare systems. Integrating mental health services and culturally sensitive health education and community engagement can help improve adherence to lifestyle recommendations.

## Introduction

Noncommunicable Diseases (NCDs) such as heart disease, diabetes, respiratory illnesses, and cancer are the leading causes of death globally, making up a 72% of the disease burden, with 80%of these premature deaths occurring in low- and middle-income countries (LMICs) [1]. As per one example of these countries, the WHO NCDs progress monitor report found that the prevalence of deaths from NCDs in Ethiopia amounted to 43%, accounting 271,300 per year [1]. The COVID-19 pandemic has had a profound impact on public health worldwide, especially on individuals with NCDs [2,3]. Beyond increasing the risk of severe COVID-19 complications, the pandemic disrupted healthcare services worldwide, including those essential for managing NCDs [4,5]. Measures such as lockdowns, resource reallocation, and reduced mobility created barriers to accessing medical services, leading to delayed treatments, reduced routine care, and increased psychological distress [6,7]. These barriers, including stress and restricted mobility, significantly impacted adherence to lifestyle recommendations such as maintaining a healthy diet, engaging in physical activity, avoiding tobacco, and reducing alcohol consumption [8,9].

People living with NCDs have faced even greater challenges during the pandemic due to compromised immune systems and difficulty accessing consistent care [10]. Moreover, pandemic-related measures, such as lockdowns and social distancing, have also significantly influenced health behaviors, leading to reduced physical activity, poor dietary habits, increased tobacco use, and elevated alcohol consumption [7,11]. One of the behaviours that has become a major concern is heavy episodic drinking (HED), also known as binge drinking. HED is having at least 60 grams or more of alcohol (corresponds approximately to 6 standard alcoholic drinks) on at least one occasion in the past 30 days [12], which can increase the risk of heart problems, liver disease, and other health complications [4].

During the pandemic, alcohol consumption increased in some countries, largely driven by stress, anxiety, and loneliness [6]. For example, a study reported that alcohol consumption increased by 21% among adults during the first year of the pandemic [6]. For individuals with NCDs, this behavior has compounded existing health risks, increasing the likelihood of cardiovascular problems, liver damage, weakened immune response, and worsened outcomes. The psychological burden of the pandemic, including fear of infection, economic instability, and isolation, has exacerbated these challenges for vulnerable groups [13,14]. In Ethiopia, alcohol use is culturally accepted, often linked to traditional events and social gatherings, which has contributed to rising consumption rates and increased health concerns for people with chronic illnesses [15,16]. This cultural acceptance can make alcohol consumption a normalized part of social life, which can contribute to increased consumption among individuals, including those with NCDs. A systematic review of 6 studies conducted in Ethiopia identified the prevalence of 12.4% to 21.1%, raising concerns about its impact on individuals with chronic illnesses [15]. Reports suggest that the availability of alcoholic beverages and the number of alcohol manufacturers have also risen in Ethiopia, further normalizing drinking behaviours [16].

This study aims to assess adherence to healthy lifestyle recommendations, with a particular focus on heavy episodic drinking among patients with NCDs during the COVID-19 pandemic in Arba Minch, Ethiopia. Understanding the prevalence of HED and identifying associated factors within this population is essential for developing targeted interventions, such as community-based alcohol reduction programs, mental health support services, and improved healthcare access. These interventions are critical to mitigating the negative health effects of alcohol use and enhancing the management of NCDs. The insights gained from this study will inform the development of strategies that promote healthier lifestyles and improve care outcomes for vulnerable populations.

## Methodology

### Study design and setting

A hospital-based cross-sectional study was conducted among 310 patients with noncommunicable diseases (NCDs) at Arba Minch General Hospital (AMGH) in Arba Minch, Ethiopia, from March 1 to April 30, 2022. The hospital is located in a demographically diverse area, representing various ethnic groups, socioeconomic statuses, languages, and religions (such as Muslim, Orthodox, and Protestant communities), making it representative of the general population within a developing country. AMGH was selected as the primary data collection site due to its high patient flow, range of services, and logistical feasibility.

### Sample size determination

The necessary sample size was calculated using the single population proportion formula, n= (Z _α/2_)^2^ * p(1−p)/d^2^ [17], where: Z is the z-value corresponding to a 95% confidence level (Z=1.96), p is the expected prevalence of the key outcome (in this case, chronic disease prevalence among the study population), d is the margin of error (set at 5%, or 0.05). This approach was chosen because the primary objective was to estimate the prevalence of heavy episodic drinking among NCD patients and following the recommendation of WHO [18] and other similar studies [19]. In addition, the formula is appropriate for estimating a single proportion with a specified level of precision.

The prevalence of HED among adults in the Arba Minch Health and Demographic Surveillance Site was 13.7%, which initially resulted in a calculated sample size of 182 participants. However, given the inclusion of multiple study variables, the prevalence of chronic disease in the region (29.7%) was used to derive a larger and more representative sample size for robust statistical analysis [20]. Based on this adjusted prevalence, the final calculated sample size was 320 participants. Finally 320 participants approached for data collection and 310 completed the interview.

### Inclusion and exclusion criteria

#### Inclusion criteria.

- All patients diagnosed with chronic diseases who had a scheduled follow-up visit at the selected hospital during data collection period were eligible for inclusion.

#### Exclusion criteria.

- Patients newly diagnosed with a chronic disease who had not yet attended a follow-up visit.
- Patients who declined to participate.
- Individuals under 18 years of age.
- Adults with severe illness or diagnosed mental illnesses, who were deemed too ill to participate by healthcare professionals.

### Data collection process

Information about the study was provided to patients using posters displayed at the hospital entrance, and patients were informed by the gatekeeper at Outpatient Department 5 (OPD5). Invitations to participate, along with a participant information leaflet and consent form, were distributed to randomly selected follow-up patients in OPD5. Randomization was performed using the lottery method, with a list of registered follow-up patients serving as the sampling frame. Random numbers were generated using the OpenEpi Random Program (www.openepi.com). When a selected patient was ineligible or unable to participate, the next patient in the sequence was invited. Participants who agreed to join the study completed the consent form, sealed it in an envelope, and submitted it to a designated collection box at OPD5.

Data collection involved face-to-face interviews and anthropometric measurements, conducted in a controlled and standardized environment in the second (back) room of OPD5. The OPD5 area includes two separate rooms—one for patient care and the other for general use—to ensure a comfortable environment for interviews.

### Data collection instruments

The data was collected using the WHO Stepwise Approach to Chronic Disease Risk Factor Surveillance (STEPS) tool. The tool has three steps:

- Step I (questionnaire),
- Step II (anthropometric measurements), and
- Step III (biochemical measurements, which was not included in this study).

Step I collected information on sociodemographic data, type of NCD, and behavioural factors such as alcohol and tobacco use, exercise, and dietary habits. Step II included anthropometric measurements (e.g., weight, height, blood pressure), which were conducted using specialized tools (digital blood pressure apparatus, height and weight scale) in accordance with WHO guidelines [18]. Additional questions assessed the impact of COVID-19 on NCD-related factors, mental health, and disruptions in healthcare. The tool was tested and validated in the previous study conducted in the study area at Arba Minch HDSS [21]. In addition, a pretest was done on 5% (n=15) of the sample, and modifications were made to the tools based on this.

For assessing the impact of the pandemic on mental health, anxiety symptoms were assessed through a 5-item Coronavirus Anxiety Scale (CAS) [22]. Participants were asked, “How often have you experienced the following activities over the last 2 weeks?”. The CAS-5 score is calculated by assigning scores of 0, 1, 2, 3, and 4, to the response categories of “not at all”, “rare, less than a day or two,” “several days,” “more than 7 days,” and “nearly every day over the last two weeks,” respectively, and then adding together the scores for the five questions to get final CAS score. Various studies demonstrated that CAS is a valid and sufficient tool to measure anxiety related to COVID-19 [22,23]. The reliability of the tool was assessed, yielding the Cronbach’s alpha =.839, which indicates good internal consistency of the scale. The CAS has been validated in previous research studies, demonstrating its reliability (α = 0.92), and validity with 76% sensitivity and 90% specificity in measuring anxiety related to COVID-19 [22].

### Statistical analysis method

The data analysis process involved both descriptive and inferential statistics. Descriptive statistics involved computations, recoding of variables, and calculations of frequencies and proportions for categorical variables. To address the issue of cell counts less than five, data were merged when the number of participants in specific categories was too small, following recommendations from the World Health Organization (WHO) and relevant research guidelines. For educational status, data were initially collected in five categories: illiterate, can read and write, primary education, secondary and preparatory education, and diplomas and above. These were later merged into three broader categories to ensure adequate representation and meaningful analysis. Similarly, marital status data was collected in six categories: unmarried, married, divorced, widowed, separated, and no response. These categories were consolidated into three groups (married, single and widowed and separated). Employment status data, which were originally collected in eight categories—farmer, daily laborer, housewife, self-employed, unemployed, government employee, pensioner, and no response—were also merged where appropriate. Please see Table 1 for new categories and operational definition. Before merging, checks were conducted to assess whether significant variations existed among the original categories. When significant differences were identified, categories were maintained separately to preserve analytical validity and interpretability. This approach ensured that the statistical analysis remained robust while addressing the challenges of small sample sizes in certain subgroups. Descriptive statistics were organized by heavy episodic drinking, stratifying sociodemographic variables, behavioral factors, and physical characteristics, disease status, anxiety levels, and healthcare disruption.

**Table 1:** The distribution of heavy episodic drinking among sociodemographic characteristics, Arba Minch General Hospital, Ethiopia, 2022 (n=310).

		Heavy episodic drinking		Total
		No (%)	Yes (%)	N (%)
Gender	Male	120 (44.3)	27 (69.2)	147 (47.4)
	Female	151 (55.7)	12 (30.8)	163 (52.6)
Age	18–34	11 (4.2)	3 (7.7)	14 (4.7)
	35–44	49 (18.8)	8 (20.5)	57 (19.1)
	45–54	75 (28.8)	11 (28.2)	86 (28.8)
	55–64	63 (24.2)	9 (23.1)	72 (24.1)
	>65	62 (23.8)	8 (20.5)	70 (23.4)
Educational status	No formal education	122 (45.0)	10 (25.6)	132 (42.6)
	Primary school	41 (15.1)	4 (10.3)	45 (14.5)
	Secondary school +	108 (39.9)	25 (64.1)	133 (42.9)
Employment status	Government employee	39 (14.4)	10 (25.6)	49 (15.8)
	Non-government employee	25 (9.2)	1 (2.6)	26 (8.4)
	Self-employed	62 (22.9)	12 (30.8)	74 (23.9)
	Unpaid	145 (53.5)	16 (41.0)	161 (51.9)
Place of residence	Urban	181 (66.8)	32 (82.1)	213 (68.7)
	Rural	90 (33.2)	7 (17.9)	97 (31.3)
Ethnic background	Gamo	167 (61.6)	25 (64.1)	192 (61.9)
	Amhara	42 (15.5)	4 (10.3)	46 (14.8)
	Zeyse	21 (7.7)	6 (15.4)	27 (8.7)
	Wolaita	18 (6.6)	1 (2.6)	19 (6.1)
	Others	23 (8.5)	3 (7.7)	26 (8.4)
Marital status	Married	211 (77.9)	32 (82.1)	243 (78.4)
	Widowed	45 (16.6)	3 (7.7)	48 (15.5)
	Single	15 (5.5)	4 (10.3)	19 (6.1)
Religion	Orthodox	140 (51.7)	27 (69.2)	167 (53.9)
	Protestant	117 (43.2)	12 (30.8)	129 (41.6)
	Muslim or other	14 (5.2)	0 (0.0)	14 (4.5)
Income status	Low	182 (67.2)	24 (61.5)	206 (66.5)
	Medium	26 (9.6)	3 (7.7)	29 (9.4)
	High	63 (23.2)	12 (30.8)	75 (24.2)

Inferential statistics included bivariate and multiple logistic regression modelling to determine associations between dependent (heavy episodic drinking) and independent variables (such as gender, education level, smoking status, healthcare disruption, and anxiety levels). Bivariate analyses identified potential predictors of HED, and variables with p≤0.2 were included in the multivariable logistic regression model using backward elimination method. Multicollinearity was assessed using variance inflation factors (VIFs), and no significant issues were identified. The final model underwent two steps, with the Omnibus Tests of Model Coefficients in the second step demonstrated a robust chi-square value of 57.301 and a highly significant p-value of less than 0.001. The Nagelkerke R Square indicated that the proportion of explained variance was 31.8%. The Hosmer and Lemeshow Test affirmed a good fit with a chi-square value of 8.584 and a non-significant p-value of.379. The model demonstrated a specificity of 98.5%, a sensitivity of 12.8%, and an overall accuracy of 87.7%. Statistical significance was determined at p ≤ 0.05. Odds ratios (COR) and adjusted odds ratios (AOR) quantified the strength of associations. Confounding variables were identified and controlled for, ensuring an accurate interpretation of the results.

### Operational definitions

**Adherence to lifestyle recommendations:** Participants who followed all recommended dietary, exercise, smoking, and alcohol consumption guidelines [19].

**Smoking-related adherence:** Participants who did not currently smoke or use smokeless tobacco products [19,24].

**Heavy episodic drinking** is having at least 60 grams or more of alcohol (corresponds approximately to 6 standard alcoholic drinks) on at least one occasion in the past 30 days [25]. (One STANDARD drink is equivalent to a can of beer (355 mL), a glass of wine (150 mL), or a shot of distilled spirits (40 mL)” [25]. Participants who did not engage in HED are called ‘adherent to alcohol’ [12].

**Sufficient vegetable and fruit intake** defined as consumption of vegetable and fruit 4–7 days per week [24].

**Physical Activity** Physical activity level categorized into low (< 600), moderate (600–3000), and high (> 3000) based on average metabolic equivalents-minutes per week) [24]. Participants who performed moderate to high physical activity are called ‘adherent to exercise’ [19]. Metabolic Equivalent Tasks (METs): METs are a standard measure of energy expenditure. For example, walking at a moderate pace for 30 minutes is equivalent to approximately 3 METs. WHO guidelines recommend a minimum of 600 METs per week for maintaining health, but higher MET levels are encouraged for additional benefits.

**BMI** **classification**: Underweight (BMI< 18.5), Normal weight (BMI 18.5–24.9), Overweight (BMI 25–29.9), and Obese (BMI≥30) [24].

**Healthcare disruption**: Patients in this category had missed or postponed at least one scheduled appointment or procedures or surgery during the pandemic, regardless of the reason for the disruption [26].

In the Employment status, **Unpaid** included peoples who had non-paid, students, housewife’s or homemakers, retired, and unemployed [24].

### Ethical considerations

Ethical approval for this research study has been obtained from all relevant institutional bodies, including the Faculty of Health Sciences Ethics Committee at Trinity College Dublin, Ireland, (Reference No 210907), the Institutional Review Board of Arba Minch College of Health Science, Ethiopia (Ref wm-24/877), as well as Arba Minch General Hospital, which, granted permission to collect data (Ref no. 11490/13). Participants were provided with detailed information about the study, including its purpose, procedures, potential risks, and benefits. Written informed consent was obtained from all participants before data collection. Participation was voluntary, and participants were assured of their right to withdraw at any time without consequence. To maintain privacy and confidentiality, all collected data were anonymized and securely stored..

## Results

### Sociodemographic characteristics of study participants

The study included 310 NCD patients who were receiving chronic illness treatment at Arba Minch General Hospital during the COVID-19 pandemic period, with a response rate of 96.8%. The mean age (SD) of the participants was 53.7 years (± 13.1), with just above half of participants being female, 163 (52.6%). See Table 1.

### Adherence to lifestyle recommendations, behavioural and disease characteristics, disease of participants

Only 16.1% [n=50, 95% CI (12.5–20.6%)] of partcipants were adherent to all recommended lifestyle modification including diet, smoking, alcohol and exercise. Two-third and one-fourth of the participants adhered to the recommended physical activity and dietary regimens, respectively. In relation to the change in alcohol consumption during the pandemic, nearly half of the participants reported increased alcohol use due to the pandemic, while 15.3% (N=13) of participants reported reduction. See Table 2.

**Table 2:** Behavioral characteristics associated with heavy episodic drinking, Arba Minch General Hospital, Ethiopia, 2022 (N=310).

		Heavy episodic drinking		Total
		No (%)	Yes (%)	N (%)
Smoking-related adherence	Adherent	266 (98.2)	32 (82.1)	298 (96.1)
	Non adherent	5 (1.8)	7 (17.9)	12 (3.9)
Adherence to diet	Adherent	73 (26.9)	1 (2.6)	74 (23.9)
	Non adherent	198 (73.1)	38 (97.4)	236 (76.1)
Current khat chewing	No	263 (97.0)	35 (89.7)	298 (96.1)
	Yes	8 (3.0)	4 (10.3)	12 (3.9)
Exercise-related adherence	Non adherent	78 (28.8)	5 (12.8)	83 (26.8)
	Moderately adherent	112 (41.3)	16 (41.0)	128 (41.3)
	Highly adherent	81 (29.9)	18 (46.2)	99 (31.9)
BMI Classification	Normal	161 (58.8)	20 (55.6)	181 (58.4)
	Underweight	26 (9.5)	3 (8.3)	29 (9.4)
	Overweight/ Obesity	87 (31.8)	13 (36.1)	100 (32.3)
Change in alcohol consumption during the COVID-19	No change	24 (49.0)	7 (19.4)	31 (36.5)
	Increased	13 (26.5)	28 (77.8)	41 (48.2)
	Decreased	12 (24.5)	1 (2.8)	13 (15.3)
History of heart attack	No	217 (80.1)	32 (82.1)	249 (80.3)
	Yes	54 (19.9)	7 (17.9)	61 (19.7)
History of raised cholesterol	No	247 (91.1)	35 (89.7)	282 (91.0)
	Yes	24 (8.9)	4 (10.3)	28 (9.0)
Comorbidity	No	184 (67.9)	24 (61.5)	208 (67.1)
	Yes	87 (32.1)	15 (38.5)	102 (32.9)
Uncontrolled HTN	No	139 (51.3)	14 (35.9)	153 (49.4)
	Yes	132 (48.7)	25 (64.1)	157 (50.6)
Healthcare disruption	No	107 (39.5)	7 (17.9)	114 (36.8)
	Yes	164 (60.5)	32 (82.1)	196 (63.2)
Coronavirus related anxiety	No	132 (48.7)	16 (41.0)	148 (47.7)
	Yes	139 (51.3)	23 (59.0)	162 (52.3)

### Impact of COVID-19 on healthcare access and mental health

The COVID-19 pandemic significantly impacted healthcare access and mental health among participants. A significant majority of NCD patients experienced, 63.2% (n=196), healthcare disruption due to the pandemic. In 52.3% [n=162, 95% CI (46.7–57.8%)] of participants reported experiencing moderate to severe anxiety symptoms related to COVID-19, as measured by the CAS. Majority of participants with HED (59.0%), had COVID-19 related anxiety. See Table 2.

### Prevalence of heavy episodic drinking (HED)

The overall prevalence of heavy episodic drinking (HED) in the study population was 12.6% [n=39/310, 95% CI (9.3% - 16.74%)]. In terms of gender distribution, a higher proportion of males (18.4%, N=27/ 147) reported HED compared to females (7.4%, N=12/163). The prevalence of HED among current drinkers was 43.3% [N=39/90, 95% CI (44.1% to 66.8%)], with the majority of males 69.2%. Regarding recent alcohol consumption within the past 12 months and within the past 30 days was 34.2% [n=106, 95% CI (29.1%- 39.6%)] and 29.0% [n=90, 95% CI (24.3–34.3)], respectively. See Table 3.

**Table 3:** The frequency of alcohol consumption by gender, Arba Minch General Hospital, Ethiopia, 2022.

		Gender		Total
		Male n (%)	Female n (%)	n (%)
Heavy episodic drinking	No	120 (81.6)	151 (92.6)	271 (87.4)
	Yes	27 (18.4)	12 (7.4)	39 (12.6)
Alcohol consumption any within the past 30 days	No	95 (64.6)	125 (76.7)	220 (71.0)
	Yes	52 (35.4)	38 (23.3)	90 (29.0)
Alcohol consumption the past 12 months	No	88 (59.9)	116 (71.2)	204 (65.8)
	Yes	59 (40.1)	47 (28.8)	106 (34.2)
Ever alcohol consumption	No	42 (28.6)	108 (66.3)	150 (48.4)
	Yes	105 (71.4)	55 (33.7)	160 (51.6)
Total		147 (100.0)	163 (100.0)	310 (100.0)

### Predictors of heavy episodic drinking

The results of the bivariate and multivariate logistic regression analysis demonstrated significant predictors of heavy episodic drinking among NCD patients. The odds of heavy episodic drinking was 2.5 times higher among males [Adjusted Odds Ratio (AOR) = 2.63, 95% CI (1.11–6.24)] compared to females, higher education levels [AOR = 2.91, 95% CI (1.11–7.58) compared to no formal education, and current tobacco use [AOR = 6.36, 95% CI (1.62–25.04)] compared to non-users. In addition, patients who experienced healthcare disruption due to COVID-19 (AOR = 3.28) and those with heightened anxiety (CAS) levels (AOR = 1.29) also emerged as significant predictors in this study. See Table 4.

**Table 4:** Univariate and Multivariate Analysis of self-reported Heavy Episodic Drinking (HED) among noncommunicable disease patients in Arba Minch General Hospital, Ethiopia, 2022, n=310.

Variables	Description	Bivariate Analysis	Multivariate Analysis
		Crude Odds Ratio (95% CI)	Adjusted Odds Ratio (95% CI)
Gender	Male	2.83 (1.38,5.82)^**^	2.63 (1.11,6.24)^*^
	Female	1	1
Educational status	No formal education	1	1
	Primary school	1.19 (0.35,4.00)	1.32 (0.34,5.10)
	secondary school +	2.82 (1.30,6.15)^**^	2.91 (1.11,7.58)^*^
Place of residence	Urban	2.27 (0.97,5.35)	1.80 (0.66,4.91)
	Rural	1	1
Current tobacco use	Yes	11.64 (3.49,38.82)^***^	6.36 (1.62,25.04)^**^
	No	1	1
Khat chewing	Yes	3.76 (1.08,13.13)^*^	1.40 (0.30,6.50)
	No	1	1
Healthcare disruption	Yes	2.98 (1.27,70)^*^	3.28 (1.16,9.26)^*^
	No	1	1
Coronavirus Anxiety Scale	Total Anxiety score	1.31 (1.11,1.54)^**^	1.29 (1.06,1.56)^*^

COR: Crude Odds Ratio; AOR: Adjusted Odds Ratio; CI: Confidence Interval;

* P- value < 0.05;

** P-value < 0.01;

*** P-value < 0.001;

## Discussion

This study investigated adherence to lifestyle recommendations, with a particular focus on heavy episodic drinking (HED), among patients with noncommunicable diseases (NCDs) during the COVID-19 pandemic in Arba Minch, Ethiopia. The findings indicated that adherence to recommended lifestyle behaviours was alarmingly low, with only 16.1% [n=50, 95% CI (12.5–20.6%)] of participants adhering to all recommended lifestyle changes during the pandemic. Specifically, 73.2% adhered to the recommended physical activity guidelines, 23.9% followed dietary recommendations, 29% reported current alcohol consumption, and 96.1% adhered to smoking-related guidelines. This study demonstrated that the prevalence of heavy episodic drinking among patients with NCDs was 12.6%, with higher proportion (18.3%) of males compared to females (7.4%).

The low adherence rate observed here is substantially lower than those reported in other Ethiopian regions, where adherence to lifestyle recommendations ranged from 23% in the central region (Addis Ababa) to 27.3% in the southern [27] and north-western regions [19,28]. These discrepancies may be attributed to differences in study populations, definitions of adherence, and socioeconomic contexts. Moreover, the pandemic’s impact on healthcare access, decreased opportunities for physical activity due to lockdowns, and restricted access to nutritious food likely contributed to the reduced adherence observed in our study. In addition, our study encompassed all patients with NCDs, whereas comparison studies focused solely on hypertensive patients, which may have influenced adherence rates [19,27,28].

The prevalence of HED (12.6%) observed in this study is higher than that reported among hypertensive patients in Addis Ababa (8.9%) [28] and northern Ethiopia (2.3%) [19]. Our findings align with the national STEPS survey in Ethiopia [29], which found a 12.4% prevalence of heavy alcohol use, as well as a community survey conducted in the Arba Minch EHSS site, which reported a prevalence of 13.7% respectively [16]. These findings indicate that the prevalence of HED among patients with NCDs in our study area is similar to that of the general population. However, given that the participants in this study already have NCDs and had been receiving treatment for at least for 6 months, it was expected to have a lower prevalence of HED compared to the general population. Because, these patients are typically more informed about the risks associated with alcohol consumption and are more likely to receive health information on the harms of substance use from the healthcare providers and other sources. The persistence of high HED rates among this population suggests that current health education and interventions are not adequately addressing the needs of these patients.

When comparing our findings to those of international studies, the prevalence of HED among individuals with NCDs in our study (12.6%) was below the global average of 18.2%, as well as rates observed in developed countries in Europe (26.4%) and America (21.3%) [1]. However, the prevalence of HED among those who reported any alcohol consumption (43.3%) was above global averages, suggesting that individuals who drink are more likely to engage in risky drinking behaviors. For example, the global prevalence of HED among alcohol consumers was 39.5%, while in Europe it was 42.6%, both of which are comparable to our findings (WHO, 2022). This underscores the necessity of targeted interventions aimed at reducing heavy drinking among current alcohol users, particularly those with underlying health issues.

The prevalence of heavy episodic drinking was found to be higher among male participants (18.4%), those with higher income and educational status, urban residents, and followers of the Orthodox religion. This trend is consistent with previous studies conducted in Ethiopia and other low- and middle-income countries, where higher socioeconomic status and urban living have been associated with increased alcohol consumption [6,16]. For instance, individuals with higher income may have greater access to alcoholic beverages, and urban residents often have more exposure to environments that normalize heavy drinking [6,30]. Moreover, Orthodox religious followers in Ethiopia may have fewer restrictions regarding alcohol use compared to other religious groups, such as Muslims, contributing to the higher prevalence observed among this group [31]. In the WHO Eastern Mediterranean Region, which are Muslim-majority countries, the prevalence of heavy episodic drinking was just 0.5%, compared to the global prevalence of 18.2% [30]. There is also an element of tradition in the types of alcohol consumed. In Ethiopia, traditional alcoholic beverages such as ‘tella’ (a type of home-brewed beer), ‘tej’ (honey wine), and ‘areki’ (distilled liquor) are commonly consumed, especially during social gatherings and cultural events [31]. This cultural acceptance and integration of alcohol into social and ceremonial occasions may further contribute to the normalization of drinking behaviours, particularly among Orthodox followers and urban residents.

Moreover, our study demonstrated that individuals with higher Coronavirus Anxiety Scale (CAS) scores were more highly engaged in HED. Similarly, several studies have reported that the pandemic was also resulted in an increased prevalence of stress, anxiety, depression, and other mental health issues [10,13,32,33], that may results in hazardous drinking and increased alcohol use among NCD patients [16,34]. Alcohol consumption is a common coping mechanism for anxiety, tension, uncertainty, and negative emotions [35], with evidence indicating increased consumption during difficult times such as disasters and pandemics [36]. This may increase the likelihood of NCD complications and have long-term health implications for individuals in these regions. It is important for policy makers and public health officials to address this issue and provide support for those struggling with mental health and alcohol use disorders [5].

Despite this, the identification of a correlation between HED and healthcare disruptions is unique finding in our study that requires careful consideration and recommendation. Because excessive alcohol consumption causes several complications in patients with non-communicable diseases that require immediate attention, treatment interruptions on top of that may result in catastrophic complications. Therefore, healthcare systems must address the impact of COVID-19 on non-communicable diseases and implement strategies to mitigate the negative consequences. By addressing healthcare disruption and related anxiety, interventions can be tailored to prevent harmful behaviors such as heavy episodic drinking. Efforts to mitigate the impact of alcohol use on NCD management should include culturally sensitive, community-based interventions that address the unique challenges faced by individuals in Ethiopia. Strengthening healthcare systems to ensure continuity of care during crises, expanding access to mental health services, and implementing public health campaigns to raise awareness about the risks of alcohol use are critical next steps. In addition, policies aimed at regulating the availability and marketing of alcohol could help reduce consumption at the population level.

Moving forward, it is essential to monitor whether the observed changes in alcohol use and healthcare access during the pandemic are sustained. Strengthening health systems and addressing gaps in care delivery will be critical to improving outcomes for individuals with NCDs. Continued research is needed to explore the long-term impact of the pandemic on lifestyle behaviours and health outcomes in Ethiopia and other similar contexts.

One of its key strengths is its ecological validity. Data were collected directly from the hospital where patients received care, ensuring the findings were based on real-world experiences. This approach made the study more relevant and reflective of the actual challenges faced by patients and healthcare providers during the pandemic. Furthermore, the study was the first in the region to assess all aspects of health among noncommunicable disease (NCD) patients, including physical, behavioral, mental, healthcare disruption, and systemic factors. This comprehensive approach provided a well-rounded understanding of the difficulties these patients have faced. In addition, the study achieved a high response rate (96.9%), which strengthens the reliability of the findings and minimizes potential response bias.

However, several limitations must be considered. The cross-sectional nature of the study limits the ability to establish causal relationships. Moreover, the reliance on self-reported data for alcohol consumption, behavioural characteristics, and health outcomes introduces the potential for recall bias and social desirability bias, as participants may underreport or overreport their behaviours. In addition, The study sample might not fully represent the broader population of individuals with NCDs during the pandemic. The recruitment process, exclusion criteria, and potential non-response bias may limit the generalizability of the results to other populations.

## Conclusion

Our study identified low adherence to recommended lifestyle modifications (7.7%) and a high prevalence of heavy episodic drinking (12.6%) among patients with NCDs during the COVID-19 pandemic in Ethiopia. The persistence of high HED rates despite ongoing treatment highlights gaps in current health education and interventions. The prevalence of HED among those consuming alcohol (43.3%) also exceeded global averages, indicating a higher risk of harmful behaviours.

These findings align with national and international studies but highlight unique cultural and social factors in Ethiopia, such as traditional alcohol consumption and acceptance among Orthodox followers. The increased prevalence of HED among males, individuals with higher income and education, and urban residents emphasizes the role of socio-demographic factors. Associations between HED, healthcare disruptions, and anxiety represent a unique finding in this study, emphasizing the critical role of mental health and healthcare access in addressing risky behaviours during crises.

To mitigate these challenges, targeted and culturally appropriate interventions are essential. Community-based alcohol reduction programs, improved healthcare access, and integrated mental health support should be prioritized. Strengthening NCD care by incorporating substance use and mental health screening can help identify at-risk patients and provide timely interventions. Similar interventions could benefit other low- and middle-income countries facing similar challenges.
